# Role of Occupational Stress and Burnout in Prevalence of Musculoskeletal Disorders Among Embassy Personnel of Foreign Countries in Iran

**DOI:** 10.5812/ircmj.9066

**Published:** 2014-05-05

**Authors:** Mashaallah Aghilinejad, Zargham Sadeghi, Amer Abdullah, Shima Sarebanha, Amir Bahrami-Ahmadi

**Affiliations:** 1 Occupational Medicine Research Center, Iran University of Medical Sciences, Tehran, IR Iran

**Keywords:** Burnout, Professional, Musculoskeletal Diseases, Occupational Stress, Embassy Personnel

## Abstract

**Background:**

Occupation is one of the major parts of our daily lives that might cause a great amount of stress. Stress and job burnout are linked together. The association between musculoskeletal disorders (MSD) and burnout syndrome as a psychosocial factor was investigated previously.

**Objectives:**

The aim of this study was to identify the role of occupational stress and burnout in musculoskeletal complaint among diplomatic employees of different embassies in Iran.

**Materials and Methods:**

In a cross-sectional study, we assessed 200 employees of the foreign countries embassies in Iran. The participants were selected randomly from all the embassy personnel. Study questionnaires were delivered to the participants and finally 161 questionnaires were returned to the researchers (response rate: 80.5%). An assessment of burnout and MSD were made using the Maslach Burnout Inventory (MBI) and Nordic questionnaires. The work place stress was measured by the work place stress questionnaire.

**Results:**

Mean occupational stress was significantly higher among embassy personnel with MSD than among the personnel without this syndrome during the preceding week (17.18 ± 3.42 and 16.06 ± 2.19, respectively; P = 0.02) and the preceding year (17.17 ± 3.11 and 16.74 ± 3.03, respectively; P < 0.01) to the study. Only smoking and occupational stress were identified as independent predictors of MSD among embassy personnel.

**Conclusions:**

It seems that association between musculoskeletal complaints and burnout syndrome was more complex than being attributed to only occupation stress. Further studies are recommended to determine other related factors to this association.

## 1. Background

Burnout is a psychological term that infers an excessive stress reaction to the prolonged exposure to occupational or professional stressors (1, 2). Burnout is often defined as the result of chronic work stress (3). If workers are unable to cope with stress factors, it might lead to multiple complications. One of the important issues of prolonged stress is burnout. The three difference dimensions of burnout are emotional exhaustion, depersonalization, and lack of personal accomplishment. Burnout has been a major topic of research throughout world. It is discussed in many studies and it has been investigated in more than 4500 studies (3).

Occupational life is one of the major parts of our daily lives that might cause a great amount of stress (4). Stress and professional burnout can be linked together. Burnout has psychological and physical symptoms including reduced energy, fatigue, weakness, chronic headache, musculoskeletal disorders (MSD), back pain, multiple physical complication, and sleep disorders (5).

The MSD are multifactorial in origin and several factors such as physical and psychological factors might contribute to their progression and persistence. Previous studies reported that sustained sitting posture during working in offices accompanied with poor ergonomic status of workplaces were among the significant causes of musculoskeletal symptoms development in office workers. The association between MSD and burnout syndrome as a psychosocial factor was investigated in previous studies (1, 5-8). Some of these studies confirmed the association between musculoskeletal complaints and burnout as a whole construct, or as a summation of its three dimensions (1, 9-11).

## 2. Objectives

Political diplomats of foreign embassies had specific work with stressful workplace and up to our searching on the literature, we did not find the same research for association between job stress, professional burnout, and MSD among diplomats and our study might be the first study in this filed. The aim of this study was to identify the role of occupational stress and burnout in musculoskeletal complaint among diplomatic employees of different embassies in Iran.

## 3. Materials and Methods

### 3.1. Study Population

In this cross-sectional study, we assessed the personnel of the foreign countries embassies in Tehran, Iran. All of the embassies personal in any part of foreign embassies were eligible for participating. The personal with bone fractures as well as systemic or tumoral bone disorders were excluded. According to the sample size formula (P = 0.5 and d = 0.05) at least 50 individuals would be needed. We prepared a list of foreign embassies in Iran and among them, due to availability of study population and without need for more expenditure on larger sample size, we randomly selected 200 individuals as study participants. Study questionnaire were distributed among the participants and finally 161 questionnaires were returned to the researchers (response rate: 80.5%). Present study was approved by Ethical Committee of Iran University of Medical Sciences and informs consents were signed by all of the participants. 

### 3.2. Data Gathering Instruments

In this study, basal variables of embassy personnel including their age, gender, education, body mass index (BMI), marital status, smoking, and past medical history were collected via study check list. The professional burnout was assessed by the Maslach Burnout Inventory (MBI). This scale consists of 19 questions relating to the three components of burnout including personal, work-related, and client-related burnout. Personal burnout is a state of prolonged physical and psychological exhaustion and is assessed by six questions. Work-related burnout is a state of prolonged physical and psychological exhaustion, which is perceived as related to the person’s work, assessed by five questions. Client-related burnout is a state of prolonged physical and psychological exhaustion, which is perceived as related to the person’s work with client, is measured using six questions. The items were scored using a five-point scale from zero to 100. The MBI had been used in the previous studies for assessment of burnout in several fields such as health care (12-14) as well as teachers and students (15, 16).

Data of musculoskeletal complaints were gathered by means of Standardized Nordic Self-Reporting Questionnaire. This questionnaire includes questions such as age, duration of occupation as a worker, weight of carried loads, daily working hours, and musculoskeletal complaints in each of the following body regions: neck, shoulder, elbow, wrist/hand, upper back, lumbar, one or both hips/thighs, one or both knees, and one or both ankle/feet. Data on daily working hours were measured by considering the time spent in the workplace. The validity and reliability of the questionnaire has been approved in different studies. Musculoskeletal complaint was defined as pain or discomfort experienced in soft tissue of the different body regions, which had occurred at least during two to three work days in the preceding week or preceding 12 months. Moreover, the pain improvement on the weekends, vacations, and holidays had to be noted. Research team supervised all medical records and questionnaire filling. Several studies were used Nordic questionnaire as instrument of assessment of MSD prevalence among workers of different workplaces (17, 18). The workplace stress was measured by the workplace stress questionnaire. The scale was developed by the Marlin Company, North Haven, CT, USA, and the American Institute of Stress, Yonkers, NY, USA. This scale included eight parts. Responses were scored using a five-point scale ranging from zero (Never) to four (Very often). This scale was used to assess the role of occupational stress in development of other factors among workers (19).

### 3.3. Statistical Analysis

Data were presented as mean ± standard deviation for continues variables and frequency (percentage) for discreet variables. Chi squared test was used to compared demographic and qualitative variable between workers with and without musculoskeletal complaints. Quantitative variables were compared between two noted groups using independent student sample t-test. Logistic regression analysis with enter model was performed to determine independent predictors of musculoskeletal complaints among embassy personnel. Age, work history, smoking, stress score, body mass index, general health scores, as well as personal, work-related, and client-related burnout were entered into the logistic regression model. Remained variables into the model were identified as independent predictors of MSD among embassy personnel. Statistical analysis was performed in SPSS (SPSS Inc., Chicago, IL, USA). P values < 0.05 were assumed as statistically significant results.

## 4. Results

Finally, 161 questionnaires were returned to the researchers. Our participants consisted of 87 males. The mean age and work history of the study participants were 39.32 ± 6.66 and 9.43 ± 6.21 years, respectively. Most of study participants have master of science (104 participants; 64.6%) as educational degree. Mean BMI in the study population was 24.08 ± 2.88. Among study participants, 129 (80.1%) persons were married and 86 (53.4%) persons were smoker. The mean of general health scores in embassy personnel was 19.69 ± 2.12. Among embassy personnel, the mean of occupation stress was 16.74 ± 3.03 and accordingly, most of the study participants were classified as fairly low 93 (57.8%) group.

### 4.1. Musculoskeletal Complaints Prevalence in Embassy Personnel at Preceding Week and Year

Prevalence of MSD in embassy personnel during the preceding week and year in different body parts were 59.6% and 75.2%, respectively. Musculoskeletal complaints in the preceding week were most commonly reported in the neck (29.80%), the wrist(s) (26.10%), lower back (19.9%), and upper back (16.80%) consecutively; in 12-month period, these rates were most commonly at the neck (49.70%), wrist(s) (36%), lower back (34.20%), and upper back (29.20%), consecutively. In the preceding year, workers reported that musculoskeletal complaints of wrist(s) (3.7%), lower back, Knee(s), and shoulder (2.5%), consecutively, caused limitation in their function. Details of other MSD prevalence were reported in Table 2 and 3 (Table 1).

**Table 1.** Musculoskeletal Disorders Prevalence During Preceding Week and Year in Participants (n = 161) ^a^

	MSD ^b^ Prevalence During Preceding Week	MSD Prevalence During Preceding Year
**Neck**	48 (29.8)	80 (49.7)
**Wrist/hand**	42 (26.10)	58 (36)
**Lumbar**	32 (19.9)	55 (34.2)
**Upper back**	27 (16.8)	47 (29.2)
**Shoulder**	12 (6.2)	14 (8.7)
**One or both knees **	8 (5)	12 (7.5)
**One or both hips/thighs**	2 (1.2)	2 (1.2)
**Elbow**	-	2 (1.2)
**One or both ankle/feet**	-	-

^a^ Data are presented as No. (%).

^b^ Musculoskeletal disorders

**Table 2.** Mean of Subscales of Professional Burnout Among Embassy Personnel ^a,b^

Study Variables	Personal Burnout	Work Burnout	Client Burnout
**Sex**			
Male	79.90 ± 15.72	73.19 ± 14.44	76.87 ± 10.42
Female	72.18 ± 15.67	72.97 ± 13.80	75.84 ± 10.21
P value	0.27	0.92	0.53
**Marital status**			
Single	73.96 ± 16.22	72.70 ± 14.24	76.97 ± 10.32
Married	73.57 ± 15.64	72.70 ± 14.24	76.97 ± 10.37
P value	0.90	0.48	0.16
**Smoking**			
Smoker	76.36 ± 15.37	74.21 ± 15.50	76.26 ± 9.70
Non-smoker	70.56 ± 15.61	76.44 ± 11.01	71.80 ± 12.29
P value	0.019	0.28	0.96
**Work history, y**			
< 5	74.71 ± 14.80	74.92 ± 12.39	74.53 ± 6.86
5-10	74.22 ± 13.60	73.45 ± 11.12	74.46 ± 9.85
10-15	70.51 ± 21.37	69.92 ± 20.78	78.85 ± 13.01
15-20	74.40 ± 16.89	74.49 ± 18.42	80.96 ± 11.75
> 20	71.87 ± 16.77	67.86 ± 11.77	87.50 ± 8.33
P value	0.84	0.51	0.001
**Age groups, y**			
< 29	67.86 ± 18.74	76.53 ± 11.82	76.19 ± 14.77
30-39	73.91 ± 15.06	74.03 ± 12.01	75.13 ± 7.02
40-49	74.50 ± 16.49	72.36 ± 18.16	75.69 ± 13.65
50-59	72.39 ± 17.07	67.86 ± 14.52	85.94 ± 10.53
P value	0.76	0.37	0.001
**BMI groups**			
< 20	70.83 ± 5.89	73.21 ± 2.52	72.92 ± 2.95
20-25	74.26 ± 14.89	74.94 ± 13.10	75.41 ± 9.35
25-30	73.58 ± 17.42	68.82 ± 16.31	78.86 ± 12.31
> 30	61.67 ± 73.66	66.43 ± 12.27	80.00 ± 12.97
P value	0.37	0.07	0.24

^a^ Abbreviation: BMI, body mass index.

^b^ Data are presented as mean ± SD.

**Table 3.** Comparison of Study Variables Among Embassy Personnel According Their Musculoskeletal Disorder Situation ^a, b^

Study Variables	MSD Present	No MSD	P value
**Sex**			0.38
Male	23 (57.5)	17 (52.9)	
Female	64 (42.5)	57 (47.1)	
**Marital status**			0.65
Single	9 (22.5)	23 (19)	
Married	31 (77.5)	98 (81)	
**Smoking**			0.01
Smoker	14 (35)	72 (59.5)	
Non-smoker	26 (65)	49 (40.5)	
**Work history, y**			< 0.001
< 5	12 (30)	32 (26.4)	
5-10	8 (20)	61 (50.4)	
10-15	12 (30)	14 (11.6)	
15-20	4 (10)	10 (8.3)	
> 20	4 (10)	4 (3.3)	
**Age groups, y**			< 0.001
< 29	5 (12.5)	2 (1.7)	
30-39	14 (35)	82 (67.8)	
40-49	17 (42.5)	25 (20.7)	
50-59	4 (10)	12 (9.9)	
**BMI groups**			0.03
< 20	1 (2.5)	1 (0.8)	
20-25	21 (52.5)	92 (76)	
25-30	17 (42.5)	24 (19.8)	
> 30	1 (2.5)	4 (3.4)	

^a^ Abbreviations: BMI, body mass index; MSD, musculoskeletal disorder.

^b^ Data are presented as No. (%).

### 4.2. Burnout Situation Among Embassy Personnel

According to results of MBI, mean of personal, work-related, and client-related burnout scores were 73.65 ± 15.71, 73.09 ± 14.11, and 76.40 ± 10.30, respectively. Mean of personal burnout score in smoker, those with master of science degree, and stressful embassy personnel was significantly higher than in other personnel. Mean of work-related burnout in embassy personnel with master of science degree and in stressful personnel was significantly higher than in other personnel. Mean of client-related burnout in embassy personnel with more than 20 years work history, stressful, and older than 50 years old was significantly higher than in other personnel.

### 4.3. Occupational Stress and Burnout Among Embassy Personnel With and Without Musculoskeletal Disorders

Mean of occupational stress in embassy personnel with MSD at the preceding week was significantly higher than in embassy personnel without MSD (17.18 ± 3.42 and 16.06 ± 2.19, respectively; P = 0.02). Mean of occupational stress in embassy personnel with MSD at the preceding year was significantly higher than in embassy personnel without MSD (17.17 ± 3.11 and 16.74 ± 3.03, respectively; P < 0.01). Mean of three subscales of burnout were significantly higher in embassy personnel with MSD in comparison with others.

### 4.4. Results of Logistic Regression Analysis

In our logistic regression analysis, after entering study variables including age, work history, BMI, smoking, occupational stress score, GHQ (General Health Questionnaire) score, personal, and work-related as well as client-related burnout into the model, only smoking and occupational stress score remained in our model. In other words, the impact of other study variables on prevalence of musculoskeletal complains was due to other factors and only smoking and occupational stress were known as independent predictor of MSD among embassy personnel (Table 4).

**Table 4.** Results of Regression Analysis in Embassy Personnel ^a^

	Beta	Standard Error	Significances	95.0% CI for Exp (B)	
				Upper	Lower
**Constant**	1.25	4.89	0.96	-	-
**Age**	1.11	0.089	0.20	0.93	1.32
**Work history**	0.89	0.088	0.20	0.75	1.06
**Smoking**	3.78	0.509	0.009	1.39	10.26
**Stress score**	1.37	0.124	0.01	1.08	1.75
**BMI**	0.90	0.089	0.25	0.76	1.08
**Personal burnout**	0.99	0.024	0.57	0.94	1.03
**Work-related burnout**	0.97	0.025	0.16	0.92	1.01
**Client-related burnout**	0.96	0.026	0.21	0.92	1.02
**GHQ score**	1.05	0.046	0.70	0.83	1.32

^a^Abbreviations: CI, confidence interval; GHQ, general health questionnaire; BMI, body mass index.

## 5. Discussion

Finally, 161 questionnaires were returned to the researchers. Mean of personal and work-related burnout score in smoker, those who hold a master of science degree, and stressful embassy personnel were significantly higher than other personnel. Mean of client-related burnout in embassy personnel with more than 20 years work history, stressful individuals, and those older than 50 years was significantly higher than the others. Mean of three subscales of burnout were significantly higher in embassy personnel with MSD in comparison with the others. Only smoking and occupational stress were identified as independent predictor of MSD among embassy personnel.

The main purpose of the present study was to determine MSD prevalence among embassy personnel of foreign countries in Iran as well as to evaluate the association of MSD prevalence with work-related factors such as occupational stress or burnout. Psychological factors such as occupational stress and burnout had impacts on musculoskeletal complaints (20). Findings of our study showed that although stress and burnout were significantly higher in embassy personnel with MSD, occupational stress had not any mediatory role in this process. In their study on different work groups, Kjellbberg and Wadman reported that stress in workers differed from burnout, although both of them were closely related to each other (20). Similar findings were reported in Maslach and Schaufeli study (21). 

Burnout is related to loss of energy and might occur in both physical and psychological part of human life. People who experience burnout most of the times, progressively loss their idealism, energy, and purpose (22). In some studies, burnout concept was defined in three main parts: emotional exhaustion, depersonalization, and lack of personal and professional completion (22, 23). Results of binary logistic regression among study variables of the present study showed that most of the suspected factors such as age, sex, work history, and other factors were dependent predictor of MSD in the workers. On the other hand, some other and non-related factors were effective in burnout development among workers. Our study results were similar to the results of the study by Larsman et al. in which few percentage of MSD cases were related to the occupational stress and professional burnout (24). Several previous studies confirmed the association of burnout syndrome with the presence and development of MSD among workers (5-8, 25).

It seems that association between musculoskeletal complaints and burnout syndrome was more complex than being attributed to occupation stress only. Musculoskeletal complaints were related to muscular tension, one of the main findings in workers with high stress level. Larsman and Hanse reported that low social support and psychological stimuli were associated with increasing risk of neck, shoulder, and low back pain (26).

Embassy personnel are the professional workers who spend most of their time in embassies in the several countries with different socioeconomically and cultural conditions. They possibly confront with many stressors and it might lead to burnout development. According to study findings for assessment and control of burnout in embassy personnel, besides psychological factors such as occupational stress, we must pay more attention to other mediated factors such as work environment, ergonomic situation, and different cultural conditions. 

Strength of our study was its study population. According to our searching in the literature, this study we did not find study that evaluate role of burnout or other psychological factors on MSD development among embassy personnel up to the date of performing. Present study had some limitations; firstly, we only selected some embassy personnel due to security and political considerations. Secondly, burnout is a psychological concept and its measurement tools had to be defined more clearly to the participants to increase study accuracy. Thirdly, embassy personnel of several countries had social and economic differences; hence, it seems that stratified sampling method is a better sampling method for the future studies.
